# Oral Administration of *Porphyromonas gingivalis*, a Major Pathogen of Chronic Periodontitis, Promotes Resistance to Paclitaxel in Mouse Xenografts of Oral Squamous Cell Carcinoma

**DOI:** 10.3390/ijms20102494

**Published:** 2019-05-21

**Authors:** Jae Min Song, Bok Hee Woo, Ji Hye Lee, Sanggyeong Yoon, Youngseuk Cho, Yong-Deok Kim, Hae Ryoun Park

**Affiliations:** 1 Department of Oral and Maxillofacial Surgery, School of Dentistry, Pusan National University, Yangsan 50612, Korea; songjm@pusan.ac.kr; 2 Department of Oral Pathology, School of Dentistry, Pusan National University, Yangsan 50612, Korea; vokiwoo@pusan.ac.kr (B.H.W.); jihyelee@pusan.ac.kr (J.H.L.); 3 Periodontal Disease Signaling Network Research Center, School of Dentistry, Pusan National University, Yangsan 50612, Korea; 4 Department of Statistics, College of Natural Science, Pusan National University, Busan 46241, Korea; tkd_rud@naver.com (S.Y.); hoys@pusan.ac.kr (Y.C.)

**Keywords:** *Porphyromonas gingivalis*, chronic periodontitis, oral cancer, chemoresistance, paclitaxel, cytokine, ibuprofen

## Abstract

Chemotherapy is not a first-line therapy for oral squamous cell carcinoma (OSCC), which is the most common type of oral cancer, because most OSCC shows resistance to chemotherapeutic reagents. Inflammatory signals are suggested to be associated with chemoresistance as well as carcinogenesis in many different cancers, and thus chronic periodontitis, the most common chronic inflammatory disease of the oral cavity, could modulate responsiveness to chemotherapeutic agents used against oral cancer. This study was performed to define the role of chronic periodontitis in oral cancer progression and to determine the responsiveness of oral cancer to a chemotherapeutic reagent. First, we quantified the tumor growth rate and changes in serum cytokine profiles of mice administered *Porphyromonas gingivalis*, a major pathogen of chronic periodontitis. Compared with uninfected mice, the mice that were chronically administered *P. gingivalis* showed increased resistance to paclitaxel and a decreased tumor growth rate. In addition, *P. gingivalis*-treated mice exhibited higher serum levels of interleukin-6 (IL-6) than uninfected mice. Furthermore, the sensitivity of tumor xenografts to paclitaxel in mice administered *P. gingivalis* was dramatically increased when the mice were administered ibuprofen, an anti-inflammatory drug which supports the modulatory effect of periodontal pathogen-induced inflammation in chemoresistance.

## 1. Introduction

During recent decades, knowledge of the correlation between inflammation and cancer has increased. Among the large amount of information indicating this relationship, the importance of inflammatory mediators has been recently emphasized in the development of resistance to chemotherapeutic agents and tumor progression [1,2]. Studies that investigated this association have revealed that cancer cells, which are continuously exposed to inflammatory signals, display chemoresistance and increased cancer cell aggression through the acquisition of stem cell properties and/or epithelial–mesenchymal transition [3,4,5,6]. Although numerous in vitro experimental studies support the hypothesis that inflammatory signals from the tumor microenvironment modulate the responsiveness of tumor cells in various types of cancer, in vivo studies, which prove the role of inflammation in the development of chemoresistance, are not conclusive [3,7,8].

In addition, it is well known that there is no effective chemotherapeutic agent for oral squamous cell carcinoma (OSCC), the most common cancer of the oral cavity, despite the striking development of numerous effective regimens for a variety of cancers, such as vincristine for leukemia and paclitaxel for lung cancer [9,10]. Most chemotherapeutic agents are usually utilized as an adjuvant therapy for oral cancer treatment to decrease the size of the tumor mass before surgery because oral cancer is rarely cured by chemotherapy and can even develop resistance to therapy [11,12,13]. Accordingly, clinicians have no choice but to perform surgery for oral cancer, and this modality results in cosmetic and functional problems that include surgical defects and limited curative effects. To improve the prognosis of oral cancer and to diminish undesirable sequelae after surgery, effective chemotherapy regimens for OSCC must be developed. These treatments could be developed with a thorough understanding of oral carcinogenesis and the factors or mechanisms that induce resistance of OSCC cells to therapeutic agents [14,15]. Among the numerous factors that can complicate the response of oral cancer to chemotherapy, infection of cancer cells with periodontitis-related microbial pathogens and the resultant inflammatory signals could be considered primarily because chronic periodontitis is the most common chronic inflammatory disease of the oral cavity and is closely associated with oral cancer [15,16,17]. In addition, both chronic periodontitis and OSCC are diseases of elderly people; the incidence rates of chronic periodontitis and OSCC increase with age, and these diseases usually affect people older than 40 years. Accordingly, it is probable that OSCC patients also have chronic periodontitis, and thus, the biological behaviors of oral cancer, including sensitivity to chemotherapy, could be modulated by signals from chronic periodontitis. Most previous studies on periodontitis focused on the regenerative capacity of and/or the material that can be applied to the damaged periodontal tissue rather than its association with oral cancer [18,19]. Recently, epidemiological and experimental studies investigating the effects of chronic periodontitis on oral cancer have been performed and reported a close correlation between them [5,16,20,21,22,23]. However, studies on the role of chronic periodontitis in the sensitivity of oral cancer to chemotherapeutic agents are limited. In vivo studies investigating the efficacy of chemotherapeutic agents under the influence of periodontal pathogens are especially rare. In this study, we observed the effect of chronic periodontitis on the tumor growth rate, paclitaxel sensitivity, and serum cytokine profile of an OSCC xenograft model to define the association between chronic periodontitis and oral cancer as well as the role of chronic periodontitis in oral cancer progression.

## 2. Results

### 2.1. Oral Administration of P. gingivalis Reduces the Effect of Paclitaxel on OSCC Xenografts

**Figure 1:**
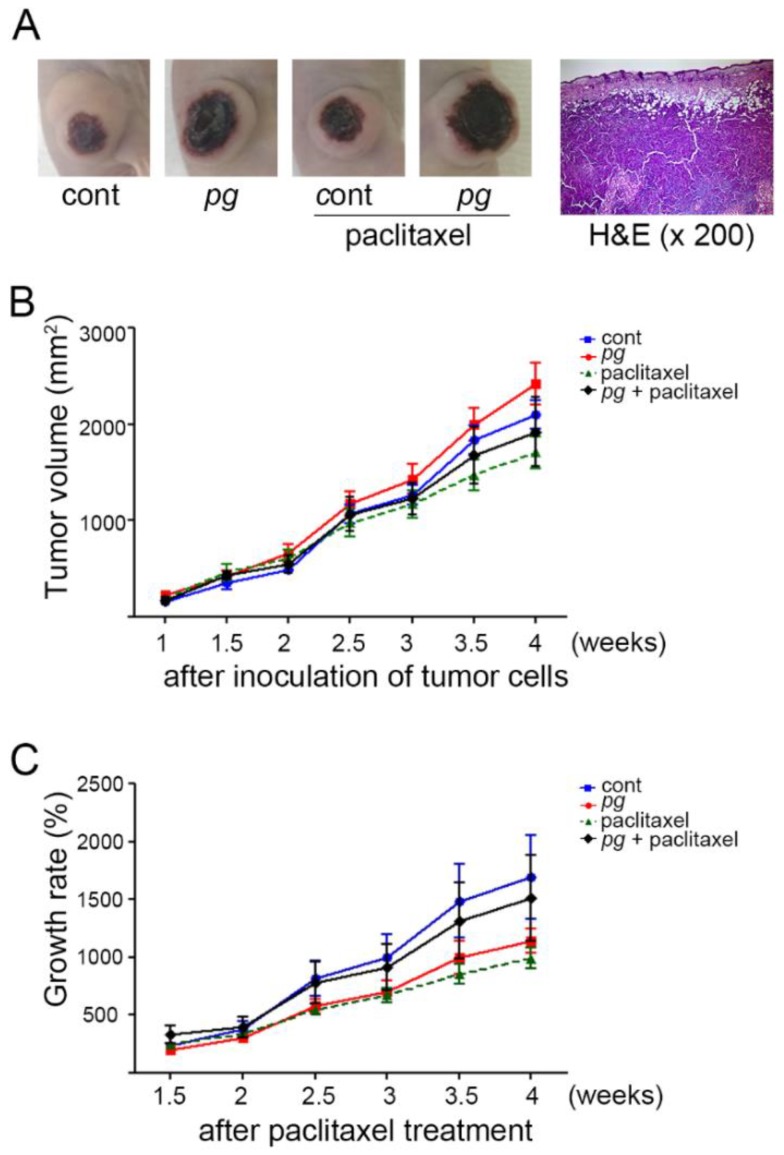
Comparisons of the tumor volume and growth rate between *P. gingivalis*-treated and/or paclitaxel-treated mice. (**A**) Photographs of OSCC xenografts in mice and representative images of H & E-stained sections of tumor masses (×200 magnification). Tumor volumes (**B**) were measured twice per week after the subcutaneous implantation of tumor cells, and the growth rate of the tumor masses (**C**) was calculated. Data are presented as the mean ± standard deviation.

To mimic the presence of chronic periodontitis, *P. gingivalis* was orally administered to mice with tumor xenografts. The reason that oral administration of *P. gingivalis* is used to induce experimental periodontitis is described below. First, though periodontitis is a multifactorial and polymicrobial disease, studies have reported that *P. gingivalis* is strongly involved in the etiology of chronic periodontitis. Second, it has been reported that oral administration of *P. gingivalis* could induce experimental periodontitis in mice [24]. Third, among numerous periodontal pathogens, *P. gingivalis* has been reported to be intimately associated with cancer progression [20,22,25]. Our previous study showed that the growth of the tumor mass was slower in mice grafted with OSCC cells that were repeatedly and sustainedly infected with *P. gingivalis* than mice grafted with uninfected OSCC cells [22]. To further clarify whether the presence of *P. gingivalis* in the tumor microenvironment also contributes to the retarded growth of OSCC, tumor xenografts were established using the OSCC line OSC-20 on the backs of mice. Then, the mice were orally administered phosphate-buffered saline (PBS) or *P. gingivalis* using Zonde, a feeding needle for mice. Tumor volume was measured whenever *P. gingivalis* was orally administered starting 7 days (1 w) after the subcutaneous injection of the OSC-20 cells. Thirty-five days after inoculation, the mice were sacrificed, and the tumor masses were excised, sectioned, and stained with H&E. The sections exhibited the histopathological morphology of moderately differentiated squamous cell carcinoma, with prominent central necrosis (Figure 1A). There was no definitive difference in histopathological grade or morphological characteristics between the *P. gingivalis* administration and the paclitaxel treatment. The tumor volumes of the *P. gingivalis*-treated mice were slightly larger than those of untreated control mice, but this difference was not significant (Figure 1B). Although this difference was statistically nonsignificant, the tumor xenografts of *P. gingivalis*-treated mice displayed a much slower growth rate than those of untreated mice, indicating that chronic infection with *P. gingivalis* does not promote tumor growth and even restricts growth potential in a xenograft mouse model of OSCC. Furthermore, to define the effect of *P. gingivalis* infection on paclitaxel responsiveness, *P. gingivalis*-treated and untreated mice were administered paclitaxel (10 mg/kg) via intraperitoneal injection 14 days after tumor inoculation, and the paclitaxel treatment continued twice per week for a 4-week period. In response to paclitaxel, *P. gingivalis*-treated mice displayed a much higher growth rate as well as a larger tumor volume than untreated mice, and the growth rate of *P. gingivalis*-treated mice with paclitaxel treatment was increased compared with that of *P. gingivalis*-treated mice without paclitaxel treatment, implying that paclitaxel stimulates tumor growth and the resultant inflammatory condition under the condition of *P. gingivalis* infection (Figure 1C). Considering that the administration of *P. gingivalis* could slow the growth of OSCC xenografts, these refractory results strengthen the notion that OSCC is resistant to paclitaxel in *P. gingivalis*-treated mice.

### 2.2. Increase in Serum IL-6 Level with P. gingivalis Administration in Mice

**Figure 2:**
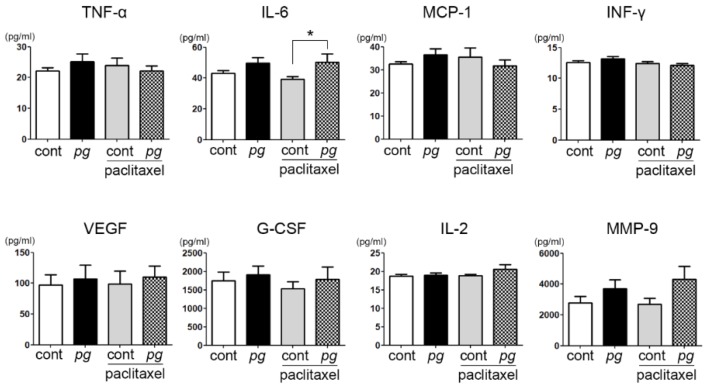
Comparisons of the serum cytokines in tumor xenografts from mice that were administered *P. gingivalis* and/or paclitaxel. Data are presented as the mean ± standard deviation. Significance was assessed using ANOVA. * *P* < 0.05.

To define the correlation between inflammatory mediators and tumor growth and/or resistance to paclitaxel, serum levels of various inflammatory factors of *P. gingivalis*-treated mice with or without paclitaxel treatment were compared with untreated and/or paclitaxel-treated mice. The serum level of IL-6 was significantly different between the control mice and *P. gingivalis*-treated and/or paclitaxel-treated mice, whereas the other inflammatory factors showed no significant differences among the groups. In particular, the mice administered both *P. gingivalis* and paclitaxel exhibited the highest level of serum IL-6, although the difference in serum IL-6 was insignificant as determined by post hoc analysis (Figure 2). IL-6, a multifunctional cytokine, is known to be associated with many different biological activities of cancer cells, including the inhibition of apoptosis and epithelial–mesenchymal transition. These characteristics of IL-6 are related to drug resistance, and their role in cancers is often reported. However, the role of IL-6 in chemoresistance is still unsettled. A study observed that an increase in IL-6 in head and neck squamous cell carcinoma was associated with cisplatin resistance.

### 2.3. Suppression of Inflammation Mitigates the Resistance of OSCC Xenografts to Paclitaxel in P. gingivalis-Treated Mice

**Figure 3:**
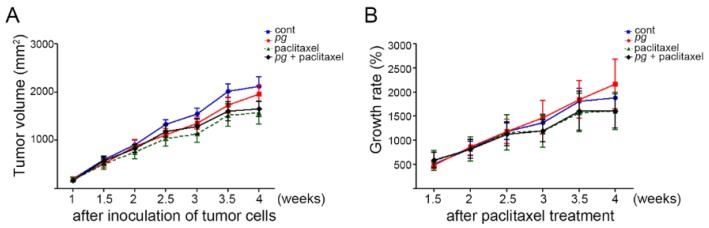
Comparisons of tumor volume and growth rate between *P. gingivalis*-treated and/or paclitaxel-treated mice administered ibuprofen. Tumor volumes (**A**) were measured twice per week after the subcutaneous implantation of tumor cells, and ibuprofen was administered in drinking water. The growth rate of the tumor masses (**B**) was calculated. Data are presented as the mean ± standard deviation. Significance was assessed using ANOVA. * *P* < 0.05.

To further examine whether the administration of *P. gingivalis* and its resultant inflammatory reaction are involved in the retarded tumor growth and paclitaxel resistance of OSCC, ibuprofen, one of the representative NSAIDs, was administered daily in drinking water to mice. In contrast to the above results (Figure 1), in the presence of ibuprofen, the tumor growth rates of *P. gingivalis*-treated mice and untreated mice were almost the same, and the tumor growth rate of *P. gingivalis*-treated mice even increased at the last measurement before sacrifice compared with that of the untreated mice (Figure 3), suggesting that ibuprofen relieved the suppression of tumor growth in *P. gingivalis*-treated mice. In addition, we investigated the response of tumor xenografts to paclitaxel in mice treated with ibuprofen. In contrast to the above observation that *P. gingivalis*- and paclitaxel-treated mice showed a much higher growth rate than untreated and paclitaxel-treated mice, in the mice that were given ibuprofen, *P. gingivalis* administration did not result in the resistance of tumor xenografts to paclitaxel, suggesting that *P. gingivalis* infection and the resultant inflammatory reactions contribute to the development of paclitaxel resistance and that paclitaxel resistance could be modified by anti-inflammatory agents.

### 2.4. Prophylactic Use of Ibuprofen Significantly Suppresses Serum Levels of MCP-1 While Increasing VEGF and MMP-9 Expression

**Figure 4:**
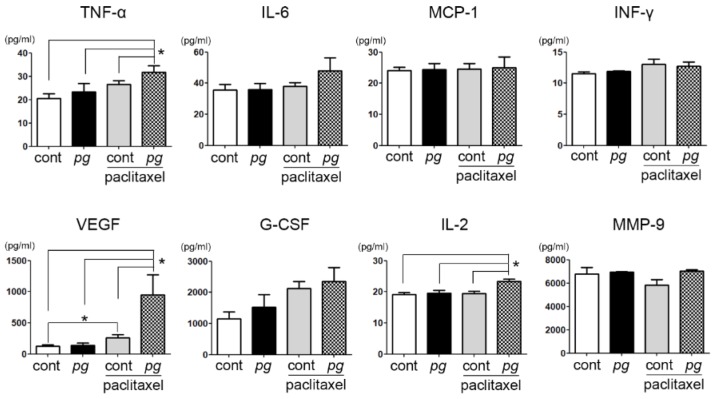
Comparisons of the serum cytokines in ibuprofen-treated mice that were administered *P. gingivalis* and/or paclitaxel. Data are presented as the mean ± standard deviation. Significance was assessed using ANOVA. * *P* < 0.05.

Under the influence of ibuprofen, no significant difference was observed in the serum IL-6 level among the groups. Considering the significant difference in the serum level of IL-6 between mice treated with paclitaxel only and those administered *P. gingivalis* and paclitaxel (Figure 2), this result implies that the effect of IL-6 on paclitaxel resistance was weakened. Moreover, significant increases in a few inflammatory mediators—such as TNF-α, VEGF, and IL-2—were observed in paclitaxel-treated mice, especially with *P. gingivalis* administration. TNF-α is known to play key roles in the cytokine network and to activate the apoptosis pathway. In this study, the sensitivity of tumor xenografts to paclitaxel was increased as TNF-α levels increased, suggesting that TNF-α may function in tumor immune surveillance. In addition, the increase in IL-2 in this study implies that the role of IL-2 in the chemoresistance modulation of oral cancer cells that accompanies chronic periodontitis should be further investigated. Over the previous three decades, IL-2 was identified and studied as a candidate for cancer immunotherapy, but its function in oral cancer progression and/or treatment has not been studied. Interestingly, the difference in the serum VEGF level of between the *P. gingivalis*-treated mice administered paclitaxel and the other groups was substantially higher than the differences in other inflammatory mediators (Figure 4). The mechanism underlying this increase in VEGF with the combined administration of *P. gingivalis* and paclitaxel is not clear; however, we suspect that the reduced paclitaxel resistance in mice administered ibuprofen may be due to the increase in VEGF, which can help deliver paclitaxel to the tumor mass.

Next, we compared the serum levels of inflammatory mediators in response to ibuprofen administration (Table 1). The level of MCP-1/CCL2, a potent chemotactic factor in monocytes, was distinctly decreased with ibuprofen administration, while the other mediators did not prominently decrease in response to ibuprofen. Additionally, ibuprofen administration significantly increased TNF-α and IL-2 levels in mice administered *P. gingivalis* and paclitaxel, suggesting that recovery of paclitaxel sensitivity may be partially dependent on the roles of TNF-α and IL-2. In addition, VEGF was increased in the presence of ibuprofen, especially in paclitaxel-treated and/or *P. gingivalis*-treated mice. MMP-9 levels were also increased in all groups in response to ibuprofen treatment by more than 2-fold, similar to previously reported results in tendon cells [26].

## 3. Discussion

It is known that cancer is caused by genetic and epigenetic changes and that inflammation promotes tumor growth by accelerating factors that are related to angiogenesis, cell motility, and cell survival [2,4,14]. Although inflammation-driven tumor growth is accepted and its mechanisms are currently defined, the characteristics of oral cancer cells accompanied by chronic periodontitis can be further modified by numerous factors, including periodontal pathogens, immune cells, and inflammatory mediators [7,27,28]. In particular, the presence of microbial pathogens can complicate the response of tumor cells to microenvironmental factors by invading host cells, disturbing host cell metabolism, and modulating the host immune response [29,30]. For example, most studies report that IL-6, an inflammatory cytokines, increases the proliferation of cancer cells, while studies using live or heat-killed microbes reported mixed results of IL-6 promoting or inhibiting cell growth [31,32,33,34,35]. Considering that *P. gingivalis*-treated mice exhibited suppressed tumor growth and ibuprofen recovered this growth suppression in the present study, this periodontal pathogen and the inflammation induced by this pathogen inhibit, rather than promote, tumor growth. The suppressed growth of tumor xenografts included by *P. gingivalis* administration could be explained by two factors. First, this result may represent an attempt to suppress tumor growth by host immune surveillance. *P. gingivalis* administration activates inflammatory reactions, and inflammatory cells are recruited to the tumor microenvironment. This recruitment of inflammatory cells restricts and suppresses tumor growth as a part of tumor immunity. Second, the presence of *P. gingivalis* within cells directly suppressed the growth of tumor xenografts by modulating cytokine secretion and metabolism of the cells. In a previous study, we observed the reduced proliferation of *P. gingivalis*-infected oral cancer cells resulting from G1 arrest and autophagy and the increased secretion of various cytokines from the cells [35]. Taken together, the previous and current data suggest that chronic periodontitis may primarily contribute to suppression of tumor growth by both the direct cellular invasion of cancer cells by periodontal pathogens and the indirect inhibitory action of immune cells, though it is probable that the tumor microenvironment—such as epithelial–mesenchymal transition, cancer stemness, and metastasis—simultaneously produces factors that promote tumor progression.

In addition to growth inhibition, the present study showed that *P. gingivalis* administration conferred resistance to paclitaxel in tumor xenografts, and the role of periodontal pathogens and inflammatory cells in this chemoresistance was further supported by the increased efficacy of paclitaxel in ibuprofen-treated mice. Although resistance to chemotherapy is mainly determined by intrinsic inherent gene expression patterns, it can be acquired through alterations induced by numerous environmental factors, including inflammatory mediators. IL-6 is an inflammatory mediator well known to mediate drug resistance by inhibiting cellular apoptosis and inducing epithelial–mesenchymal transition. Our experiment also suggested that IL-6 may be the most important factor for the development of chronic periodontitis-induced chemoresistance by showing that the serum level of only IL-6 was significantly impacted by *P. gingivalis* administration and/or paclitaxel treatment and that ibuprofen reduced paclitaxel resistance and the differences in the serum IL-6 levels among groups. In addition to IL-6, the changes in other inflammatory cytokines—including IL-2, TNF-α, and VEGF—imply that numerous cytokines are intricately involved in this chemoresistance, but the precise role of these cytokines needs to be defined through further studies investigating each cytokine. In addition to these extracellular inflammatory factors involved in the chemoresistance of oral cancer, periodontal pathogen-induced intracellular alterations in OSCC may also play a role in the development of resistance to chemotherapeutic agents.

Cancer is characterized by the uncontrolled and unlimited growth of tumor cells with high replicative potential. Thus, cancer therapy drugs have primarily been developed to target actively proliferating cancer cells and have shown numerous successes in treating various kinds of cancer. However, the limited effectiveness of chemotherapeutic agents is reported in some types of cancer, including oral cancer, and relapse after chemotherapy is sometimes observed. It has been suggested that these complex results may be due to the presence of dormant or slow-cycling cells within tumors that exhibit resistance to chemotherapy-induced apoptosis [36]. We previously reported that *P. gingivalis*, a major pathogen in chronic periodontitis, induced cell cycle arrest of OSCC cells by enhancing the autophagic response, and in this study, we observed that the growth of tumor xenografts was retarded in *P. gingivalis*-treated mice [22]. We suspect that the slow growth of OSCC cells may function in decreasing the efficacy of paclitaxel in *P. gingivalis*-treated mice.

In summary, we demonstrated that the administration of *P. gingivalis* delayed tumor growth and provoked resistance to paclitaxel, which supports the close correlation between chronic periodontitis and oral cancer. In addition, this study suggests that the molecular mechanisms responsible for this resistance may be related to inflammatory cytokines, especially IL-6. Though the specific inflammatory mediators and the mechanism involved in this resistance cannot be determined, the finding that the prophylactic use of ibuprofen improves the resistance of OSCC to paclitaxel suggests that the combinational use of anti-inflammatory drugs could be considered to improve the efficacy of chemotherapy in oral cancer. However, this cannot be clearly concluded due to the limitations of this study, such as the lack of statistical significance and incomplete chronic periodontitis model. Although *P. gingivalis* is one of the most representative pathogens of chronic periodontitis, this disease is polymicrobial. Further research is needed to develop a strategy that sensitizes the resistant oral cancer cells that accompany chronic periodontitis.

## 4. Materials and Methods

### 4.1. Cell and Bacterial Cultures

OSC-20, a human OSCC line, was cultured in a 1:1 mixture of Dulbecco’s modified Eagle’s medium and Ham’s F-12 nutrient mixture (DME/F12; HyClone, Logan, UT, USA) supplemented with 10% fetal bovine serum (FBS; Thermo Fisher Scientific, Waltham, MA, USA), streptomycin (100 μg/mL) and penicillin (100 IU/mL) (Thermo Fisher Scientific) at 37 °C in a 5% CO2 incubator. The *P. gingivalis* strain 381 was cultured anaerobically and grown in a GAM broth (Nissui, Tokyo, Japan) that contained 5 mg/mL hemin and 5 μg/mL vitamin K at 37 °C.

### 4.2. Animals and Experimental Design

Six-week-old BALB/c male mice (ORIENT, Seongnam, Republic of Korea) weighing approximately 18–20 g were housed in a specified opportunistic pathogen-free environment at the Experimental Animal Center of Pusan National University and were used for all experiments. To establish tumor xenograft models, 2 × 10^6^ OSC-20 cells were subcutaneously injected into the backs of the nude mice. Tumor growth in the mice was monitored regularly, and 7 days after the injection of the cells, the mice had visible tumors. Then, the mice were randomly divided into four groups of six mice each. The sample size needed for statistical significance was calculated using Mead’s resource equation. In the control group of six mice, each mouse was orally administered 0.1 mL of phosphate-buffered saline (PBS). In the *P. gingivalis*-treated group of six mice, each mouse received *P. gingivalis* at a dose of 5.0 × 10^8^ CFU via oral administration. For the paclitaxel-treated group of six mice, 10 mg/kg of paclitaxel (Sigma-Aldrich, St. Louis, MO, USA) was intraperitoneally infused. In the fourth group of six mice, the mice were orally administered *P. gingivalis* and were intraperitoneally injected with paclitaxel. *P. gingivalis* was administered orally twice per week using a long metal gastric needle. Paclitaxel was injected intraperitoneally the day after the oral administration of *P. gingivalis*. Tumor volumes were determined when the mice were orally administered *P. gingivalis*. To minimize examiner variability, the measurement of the tumor masses was exclusively performed by one well-trained technician. Four weeks after infection and/or paclitaxel treatment, all the mice were euthanized, and the tumors were harvested. Serum samples were also collected and stored at −80 °C until use.

To observe the effect of anti-inflammatory action on tumor growth and responsiveness to paclitaxel in an OSCC xenograft mouse model, ibuprofen, a non-selective non-steroidal anti-inflammatory drug (NSAID), was used. Ibuprofen was purchased from a pharmacy and was dissolved in drinking water (0.1 mg/mL). All mice were permitted to drink the water and were fed ad libitum. All procedures were performed in accordance with the guidelines established by the Association of Laboratory Animal Science of Pusan National University (PNU-2016-1115, 04 Mar 2016).

### 4.3. Histopathological Analysis

Tissue samples from the tumor masses were fixed in 10% neutral-buffered formalin and embedded in paraffin. Hematoxylin and eosin (H&E)-stained sections were observed by light microscopy to examine the morphological characteristics of the tumor xenografts.

### 4.4. Multiplex Beads Assay

The levels of various cytokines in the serum of mice were measured using a MILLIPLEX Map Human Cytokine/Chemokine kit (Millipore, Billerica, MA, USA) on a Luminex 200 system (Luminex, Austin, TX, USA), which employed xMAP technology, according to the manufacturer’s instructions. Briefly, wash buffer was added into each well of a plate, after which the buffer was removed, and premixed standard cocktails and serum samples were added. Beads coupled with antibodies against tumor necrosis factor-α (TNF-α), monocyte chemoattractant protein-1 (MCP-1), vascular endothelial growth factor (VEGF), interleukin (IL)-2, IL-6, interferon-γ (IFN-γ), granulocyte colony stimulating factor (G-CSF), and matrix metalloproteinase-9 (MMP-9) were diluted in blocking buffer. Each sample was then incubated with the bead solution for 2 h at room temperature. Following one wash, a premixed detection antibody cocktail was added, and the plate was incubated for 1 h. The streptavidin–phycoerythrin mixture was added to each well, and then the beads were resuspended in PBS. The Luminex 200 platform coupled with BioRad Bio-Plex software (BioRad, Hercules, CA, USA) was used to measure the cytokine levels.

### 4.5. Statistical Analysis

Data were analyzed with SPSS v25 software package (SPSS, Chicago, IL, USA). Comparisons between groups were analyzed using ANOVA, post-hoc analysis, and Student *t*-test. No adjustments for multiple tests were applied, and all data are presented as the mean ± SD. P values less than 0.05 were considered significant.

## Figures and Tables

**Table 1:** Comparison of the cytokine profiles between ibuprofen-treated and untreated mice. Data are presented as the mean ± standard deviation. Significance was assessed using ANOVA. * *P* < 0.05; ** *P* < 0.01; *** *P* < 0.001.

**Control**	**Mean ± SD (pg/mL)**		***p* Value**
	**Ibuprofen (−)**	**Ibuprofen (+)**	
TNF-α	22.100 ± 3.665	20.625 ± 5.528	0.529
MCP-1 ***	32.450 ± 3.685	24.125 ± 2.800	<0.001
VEGF	97.150 ± 52.341	122.563 ± 72.128	0.315
IL-2	18.700 ± 1.494	19.125 ± 1.808	0.573
IL-6	43.000 ± 6.429	35.625 ± 9.768	0.101
IFN-γ *	12.600 ± 0.843	11.500 ± 0.926	0.034
G-CSF	1750.950 ± 743.944	1156.500 ± 637.470	0.092
MMP-9 ***	2767.100 ± 1407.780	6794.313 ± 1534.532	<0.001
**Pg**	**Mean ± SD (pg/mL)**		***p* Value**
	**Ibuprofen (−)**	**Ibuprofen (+)**	
TNF-α	25.200 ± 8.011	23.250 ± 10.306	0.657
MCP-1 **	36.600 ± 7.863	24.375 ± 5.553	0.006
VEGF ***	106.800 ± 71.311	138.188 ± 105.538	<0.001
IL-2	18.900 ± 2.283	19.563 ± 2.665	0.633
IL-6 *	49.850 ± 10.970	35.750 ± 11.668	0.021
IFN-γ **	13.200 ± 1.033	11.875 ± 0.354	0.006
G-CSF	1909.000 ± 750.013	1523.063 ± 1140.469	0.400
MMP-9 ***	3697.850 ± 1861.348	6952.750 ± 124.409	<0.001
**Paclitaxel**	**Mean ± SD (pg/mL)**		***p* Value**
	**Ibuprofen (−)**	**Ibuprofen (+)**	
TNF-α	24.000 ± 7.557	26.500 ± 4.375	0.420
MCP-1 *	35.550 ± 12.348	24.563 ± 5.039	0.016
VEGF **	99.100 ± 64.781	258.000 ± 161.402	0.009
IL-2	18.800 ± 1.229	19.375 ± 2.134	0.460
IL-6	39.300 ± 5.599	38.062 ± 6.774	0.408
IFN-γ	12.450 ± 0.832	13.000 ± 2.507	0.522
G-CSF	1538.350 ± 591.133	2126.875 ± 626.152	0.083
MMP-9 **	2694.600 ± 1204.875	5833.687 ± 1307.932	0.001
***Pg* + Paclitaxel**	**Mean ± SD (pg/mL)**		***p* Value**
	**Ibuprofen (−)**	**Ibuprofen (+)**	
TNF-α **	22.100 ± 5.607	31.750 ± 8.013	0.008
MCP-1	31.750 ± 8.244	25.063 ± 9.563	0.274
VEGF **	109.900 ± 57.058	951.500 ± 899.341	0.001
IL-2 *	20.600 ± 3.864	23.375 ± 2.066	0.043
IL-6	50.300 ± 17.385	47.938 ± 23.501	0.696
IFN-γ	12.100 ± 1.101	12.750 ± 1.832	0.364
G-CSF	1782.600 ± 1050.864	2348.000 ± 1268.862	0.316
MMP-9 *	4310.300 ± 2671.717	7027.625 ± 360.735	0.011

## Abbreviations

OSCC	Oral Squamous Cell Carcinoma
*P. gingivalis*	*Porphyromonas gingivalis*
IL-6	Interleukin-6
MMP	Matrix metalloproteinase
